# Detection of Pathologic Heart Murmurs Using a Piezoelectric Sensor

**DOI:** 10.3390/s21041376

**Published:** 2021-02-16

**Authors:** Kiichi Takahashi, Kyoichi Ono, Hirokazu Arai, Hiroyuki Adachi, Masato Ito, Akie Kato, Tsutomu Takahashi

**Affiliations:** 1 Department of Pediatrics, Akita University Graduate School of Medicine, 1-1-1, Hondo, Akita 010-8543, Japan; pediatr@med.akita-u.ac.jp (K.T.); cfa69550@med.akita-u.ac.jp (H.A.); d5516014@s.akita-u.ac.jp (M.I.); d5516008@s.akita-u.ac.jp (A.K.); tomy@med.akita-u.ac.jp (T.T.); 2 Department of Cell Physiology, Akita University Graduate School of Medicine, 1-1-1, Hondo, Akita 010-8543, Japan; 3 Department of Neonatology, Akita Red Cross Hospital, 222-1 Nawashirosawa Saruta Kamikitade, Akita 010-1495, Japan; arahiro@med.akita-u.ac.jp

**Keywords:** cardiac murmur, congenital heart defects, heart sound, noninvasive, piezoelectric sensor

## Abstract

This study aimed to evaluate the capability of a piezoelectric sensor to detect a heart murmur in patients with congenital heart defects. Heart sounds and murmurs were recorded using a piezoelectric sensor and an electronic stethoscope in healthy neonates (n = 9) and in neonates with systolic murmurs caused by congenital heart defects (n = 9) who were born at a hospital. Signal data were digitally filtered by high-pass filtering, and the envelope of the processed signals was calculated. The amplitudes of systolic murmurs were evaluated using the signal-to-noise ratio and compared between healthy neonates and those with congenital heart defects. In addition, the correlation between the amplitudes of systolic murmurs recorded by the piezoelectric sensor and electronic stethoscope was determined. The amplitudes of systolic murmurs detected by the piezoelectric sensor were significantly higher in neonates with congenital heart defects than in healthy neonates (*p* < 0.01). Systolic murmurs recorded by the piezoelectric sensor had a strong correlation with those recorded by the electronic stethoscope (ρ = 0.899 and *p* < 0.01, respectively). The piezoelectric sensor can detect heart murmurs objectively. Mechanical improvement and automatic analysis algorithms are expected to improve recording in the future.

## 1. Introduction

Congenital heart defects (CHDs) are the most common congenital anomalies and are present in approximately 1% of live-born infants [1]. Although it is well known that severe congenital heart disease in the neonatal period can exist without the presence of a murmur or other clinical signs, approximately one-quarter of infants with heart murmurs are diagnosed with CHDs on routine physical examination [2]. The diagnosis of CHDs in neonates is based on physical examination findings, such as heart murmurs, tachypnea, or cyanosis. The presence of heart murmurs is often the first sign of CHDs [3], and it may be the only clinical sign observed in infants [4]. However, cardiac auscultation depends on the skill of the physicians. While experienced clinicians can detect cardiac anomalies accurately by auscultation using a stethoscope alone, it is difficult for trainee physicians [5], which may lead to delayed or missed diagnoses of CHDs [6]. Other methods of detecting CHDs are electronic stethoscopes and echocardiography. However, the hard ultrasound probe and stethoscope chest piece must come in direct contact with the skin during these examinations. Moreover, patients in the neonatal intensive care unit (NICU) are usually placed in a prone position during the acute stage of their respiratory illness; examiners must change the patient’s position to supine for every examination. Since such handling may create noxious stimulation with subsequent abrupt elevations in arterial blood pressure, these methods are undesirable for extremely preterm neonates who are at a risk of intraventricular hemorrhage induced by such abrupt elevations [7]. Therefore, a simple tool that can be easily used by any physician to detect heart murmurs without handling is in high demand.

Piezoelectric sensors (PS), which capture vibrations and convert them into electrical signals, can detect heartbeats and respiratory movements [8]. These sensors have been experimentally used to detect only the heart and respiratory rates [9,10,11]. We had previously developed a PS system for the cardiorespiratory monitoring of neonates [12]. During that study, we suggested that the PS could also detect heart murmurs, although systematic analysis was not carried out. Pathologic heart murmurs, which are initiated by abnormal blood flow through defective heart valves, septa, and narrowed cardiac arteries, cause slight vibrations on the anterior chest wall of infants [13]. As these signals can be detected by placing the PS under the patients, handling can be minimized. The PS will be a useful tool to continuously monitor heart murmurs in extremely preterm neonates who are at risk of intraventricular hemorrhage induced by excessive handling. In this study, we assessed the ability of the PS to detect heart murmurs during neonatal CHD screening.

## 2. Materials and Methods

### 2.1. Subjects

Only neonates born at Akita University Hospital were included in the study. This study enrolled nine neonates who had CHDs with systolic murmurs and nine healthy neonates without any heart murmurs. No patients had diastolic murmurs during the study. Auscultation was performed by a senior pediatric cardiologist before echocardiographic diagnosis. Postnatal cardiac diagnosis was confirmed via an echocardiographic examination by a single senior pediatric cardiologist before PS data acquisition. Two patients (one with tetralogy of Fallot and one with pulmonary atresia and ventricular septal defect) were already diagnosed prenatally. Table 1 shows the clinical characteristics of patients.

### 2.2. Piezoelectric Sensor Construction

**Figure 1:**
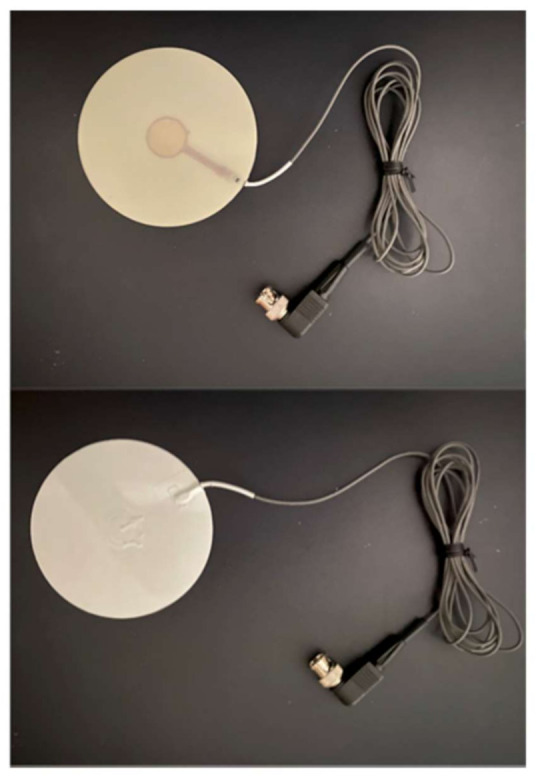
The appearance of the piezoelectric sensor (PS). The upper panel shows the bottom surface of the PS. The lower panel shows its top surface, which faces the patients. The diameter of the sensor is 100 mm. The sensor is covered with an insulation material.

The sensor developed in our previous study [12] was designed to detect mechanical vibrations caused by respiratory movements and heartbeats when the sleeping position of all neonates was routinely changed (see below). We noticed that the heartbeat signals were more marked when the neonates were placed in the prone position. Meanwhile, the detection of heartbeats was influenced by the position of the sensor, particularly in the prone position, and displacement from the best monitoring position significantly attenuated the amplitudes of the heart sounds and murmurs. Although the American Academy of Pediatrics recommends that healthy premature infants should be routinely placed in the supine position to sleep before discharge [14], infants in the NICU are often placed in a prone position during the acute stage of their illness because the prone position elevates the PaO_2_, improves thoracoabdominal synchrony, ameliorates the quality of sleep, and reduces stress behaviors [15,16,17,18]. We therefore modified the PS to detect heartbeats and thrills from the precordium. The PS is composed of a piezoelectric ceramic plate made from lead zirconate titanite (14 mm diameter) that is attached to a round copper plate (20 mm diameter) and a circular plastic plate (100 mm diameter, 0.5 mm thick) (Figure 1). The size of the plastic plate was determined in order to collect the vibrations of heartbeats.

**Figure 2:**
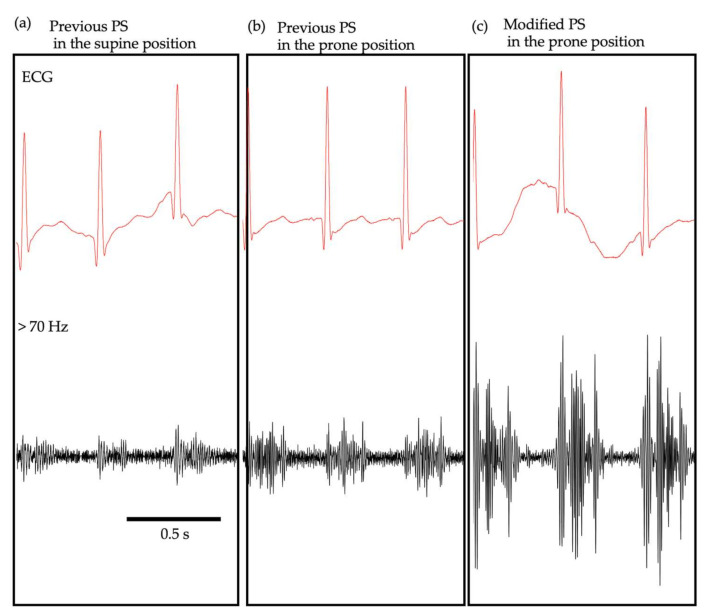
Comparison of heart sound signals and murmurs between the previous and modified PS. Simultaneous recording of ECG and the PS waveforms recorded with the previous PS in the supine (**a**) and prone (**b**) positions and the modified PS in the prone position (**c**). The PS waveforms were high-pass-filtered with a cut-off frequency of 70 Hz.

Figure 2 shows examples of the heart sound signals recorded by the previous PS in the supine (Figure 2a) and prone positions (Figure 2b) and by the modified PS in the prone position (Figure 2c). In this case, the neonate was diagnosed with a small ventricular septal defect and was diagnosed with Levine 2 systolic murmurs on auscultation by a neonatologist. The PS waveforms were high-pass-filtered with a cut-off frequency of 70 Hz to obtain the heart sound signals. The simultaneous electrocardiogram (ECG) recording enabled the identification of S1 and S2 and the systolic murmurs in all measurement conditions. It appeared that the heart sound signals and systolic murmurs were stronger in the prone position than in the supine position, when the previous PS was used. In addition, the modified PS, used in the present study, appeared superior to the previous one for detecting both the heart sounds and murmurs. When the magnitude of the systolic murmur was normalized in reference to that of the basal level (*SNR_sys_*; for explanation, see below), it was 6.65, 9.12, and 14.45 for the previous PS in the supine position, the previous PS in the prone position, and the modified PS in the prone position, respectively.

### 2.3. Data Acquisition

As we could not detect heart murmurs with the PS when the neonates were placed in the supine position in our preliminary study, in this work, we performed the measurements with neonates in the prone position. As the neonates were hospitalized, their body positions were changed to the supine, lateral, and prone positions every 3 h for routine care by a hospital nurse. To avoid excessive handling, we conducted the PS measurement when the neonates were in the prone position and did not change their positioning to perform our measurements. Moreover, to avoid the influence of position changes, we conducted the measurements at a different time from thοse for the postural changes. To examine the effect of the postural changes on the cardiovascular system, the heart rate and oxygen saturation were measured while in both the supine and prone positions. 

The PS was placed under a face towel (~5 mm thick) to avoid direct contact of the PS with the neonate’s skin. Adhesive ECG electrodes, the orogastric tube, percutaneous central venous catheter, and oxygen saturation monitor were attached to the neonates. For each patient, the PS signals were recorded for >3 min along with simultaneous three-lead ECG. The PS and ECG signals from the patients’ monitor (Dynascope DS-8100 System, Fukuda Denshi, Tokyo, Japan) were digitized at 4 kHz (PowerLab 2/26, AD Instruments, Dunedin, New Zealand) and stored on a computer using signal acquisition software (LabChart 8, AD Instruments, Dunedin, New Zealand). The PS signals were low-pass filtered with a cut-off frequency of 1 kHz (E-3201A Decade Filter, NF Electronic Instruments, Tokyo, Japan) prior to analog-to-digital conversion. On the same day as PS recording, we recorded the heart sounds using an electronic stethoscope (3M Littmann 3200 Electronic Stethoscope, 3M Health Care, St. Paul, MN, USA) for 30 s at the point of the anterior chest where the maximum amplitude of the heart sound signals was obtained when the neonates were in the supine position. The heart sound signals were converted into a WAV format. The sampling frequency of the heart sounds was 4 kHz.

### 2.4. Signal Processing

For each recording using the PS and electronic stethoscope, 10 stable consecutive beats with no obvious artifacts due to body movements were extracted. Then, the PS signals were digitally high-pass filtered with various cut-off frequencies to visualize the heart murmur and the first (S1) and second (S2) heart sounds. It has been reported that cardiac sound signal segmentation can be performed by envelope extraction algorithms, such as Shannon energy [19], Hilbert transform [20], and S-Transform [21]. In this study we simply used the Hilbert transform to calculate the envelope [*e(t)*] of the processed signals [*x(t)*], which were defined as follows:

(1) $$e\left(t\right)=\;\sqrt{x^{2}\left(t\right)+\;{\hat{x}}^{2}},$$

where $\hat{x}$(*t*) was the Hilbert transform of *x*(*t*), which was defined as:

(2) $$F\left(t\right)=\;\frac{1}{\pi }\int_{-\infty }^{\infty }\frac{x\left(\tau \right)}{t-\tau }d\tau .$$

**Figure 3:**
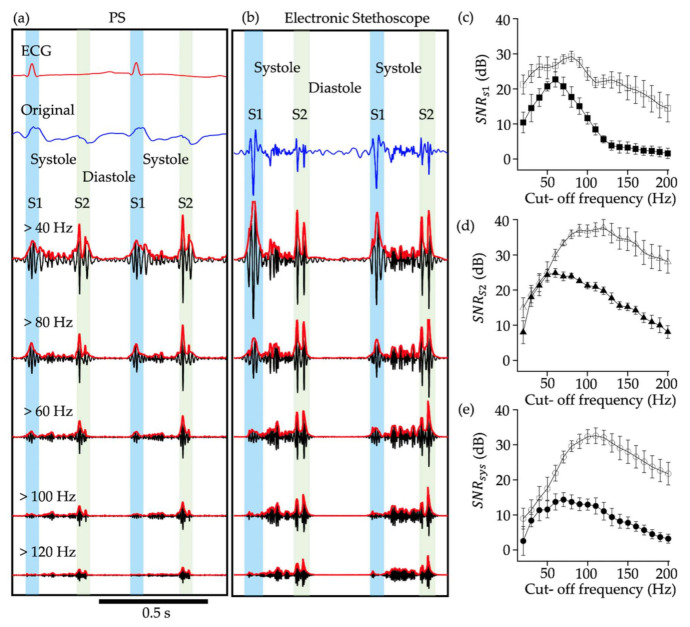
Piezoelectric sensor (PS waveforms and heart sound signals with various cut-off frequencies. (**a**) PS outputs filtered by high-pass filtering with various cut-off frequencies indicated by numerals. The first (S1) and second (S2) heart sounds were distinguished by ECG signals. The red waveform represents the envelope of the filtered signals. Electrocardiogram (ECG) signal was shown on the top and the blue wave form represents the original output. (**b**) Output of electronic stethoscope. There is no simultaneous recording ECG signal. (**c**)–(**e**) The relationship between *SNR_s1_* (**c**), *SNR_s2_* (**d**), and *SNR_sys_* (**e**) are presented. Filled and open symbols indicate the data for PS and electronic stethoscope, respectively. Each point indicates the mean and standard error of 10 consecutive heart beats. *SNR_sys_*, signal-to-noise ratio during systole; *SNR_s1_*, signal-to-noise ratio during S1; *SNR_s2_*, signal-to-noise ratio during S2

**Figure 4:**
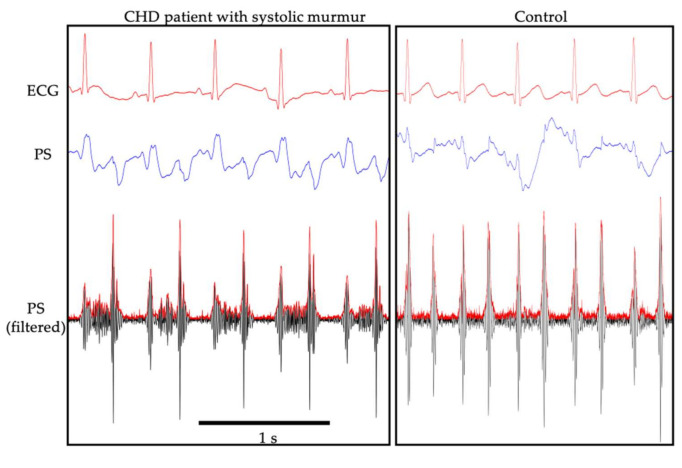
A representative recording using the piezoelectric sensor (PS) in a patient with congenital heart defects with systolic murmur (left) and that of a control (right). Electrocardiogram (ECG) signal (upper), PS output (middle), and PS output filtered by high-pass filtering (bottom) are presented.

Based on the simultaneous recording of the ECG and the filtered PS signals, S1 generated in synchronization with the R wave of the ECG and S2 located between the two R waves were clearly identified (see Figure 3 and Figure 4). Thus, the signals were divided manually into four phases of the cardiac cycle: S1, systole, S2, and diastole. As for the heart sound signals of the electronic stethoscope, S1 and S2 were identified manually by the neonatologist. The average intensities of the envelope during each phase were calculated. To evaluate the magnitude of the systolic murmur, the signal-to-noise ratio (*SNR_sys_*) was scaled by considering the amplitude of the signal during S1 and S2. Moreover, we considered the SNR during systole (*SNR_sys_*) as the signal and that during diastole as the noise. The SNRs of S1 (*SNR_s1_*), S2 (*SNR_s2_*), and *SNR_sys_* were defined as follows:

(3) $$SNR_{s1}=20\log_{10}\frac{S_{1}}{N_{dia}},$$

(4) $$SNR_{s2}=20\log_{10}\frac{S_{2}}{N_{dia}},$$

(5) $$SNR_{sys}=20\log_{10}\frac{S_{sys}}{N_{dia}},$$

where *S_1_*, *S_2_*, *S_sys_*, and *N_dia_* indicate the average amplitude of the envelope during S1, S2, systole, and diastole of 10 consecutive beats, respectively. All procedures were conducted using waveform analysis software (Igor Pro 8.03, Wavemetrics, Portland, OR, USA).

### 2.5. Statistical Analyses

Data were analyzed using SPSS Statistics (IBM Corp., Armonk, NY, USA). Paired t-tests were used to assess the differences in heart rate and oxygen saturation between the supine and prone positions. The mean *SNR_sys_* systolic murmur values in the CHD and control groups were analyzed using a Mann–Whitney U test. The correlation between *SNR_sys_* measured by the PS and that measured by an electronic stethoscope were analyzed using Spearman’s correlation method. A linear regression equation was used to describe the relationship between *SNR_sys_* measured by the PS and that measured by the electronic stethoscope. Values of *p* < 0.05 were considered significant.

### 2.6. Ethical Statement

This study protocol was approved by the Ethics Committee of Akita University Graduate School of Medicine (Ethics approval no. 1796, 2372). We provided the parents of the patients with an opportunity to opt out of the study through a poster in the NICU and posting on the website of Akita University Hospital with permission from the Ethical Review Board.

## 3. Results

We first determined the optimum cut-off frequency for detecting heart murmurs. Figure 3 shows examples of signal processing of PS waveforms (Figure 3a) and the heart sound signals of the electronic stethoscope (Figure 3b), obtained from a neonate who was diagnosed with tetralogy of Fallot and was auscultated with systolic murmurs with Levine 3 by a neonatologist. The high-pass filtered PS waveforms (Figure 3a) and the heart sound signals (Figure 3b) with various cut-off frequencies ranging from 20 to 200 Hz showed clearly that S1, S2, and the heart murmurs were clearly visualized in the outputs of the PS and the electronic stethoscope. However, the SNR was different between the PS and the electronic stethoscope and also dependent on the cut-off frequency. In general, the SNR of the electronic stethoscope was superior to that of the PS at all cut-off frequencies examined. *SNR_s1_*, *SNR_s2_*, and *SNR_sys_* of PS increased with increasing cut-off frequency, peaking at 70 Hz, and further increase of the cut-off frequency led to decreased SNR. On the other hand, the peak SNR for the electronic stethoscope was approximately 80, 130, and 120 Hz for *SNR_s1_*, *SNR_s2_*, and *SNR_sys_*, respectively. Based on the aforementioned results, the cut-off frequency of high-pass filtering was set at 70 Hz in the following experiments. 

**Figure 5:**
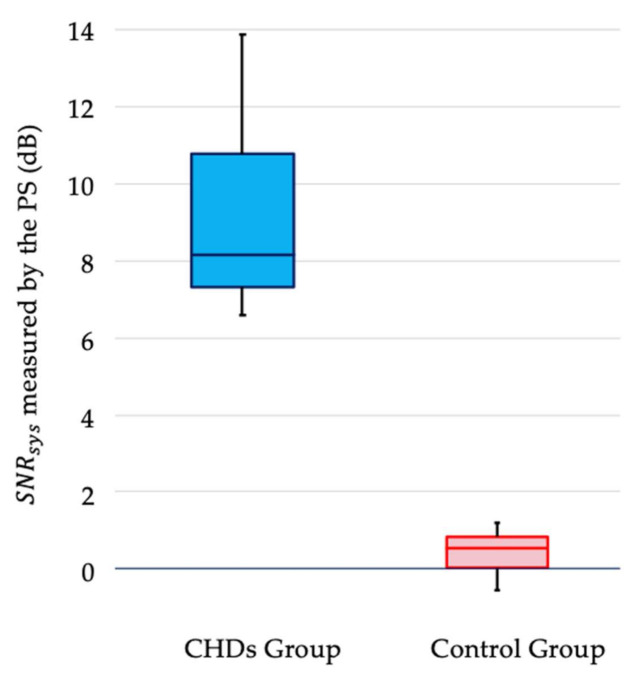
The signal-to-noise ratio (*SNR_sys_*) of a systolic murmur in nine patients with congenital heart defects (CHDs) and nine healthy neonates. The *SNR_sys_* of systolic murmur was significantly higher in patients with CHDs than in healthy neonates (*p* < 0.01).

We could detect heart sounds and systolic heart murmurs graphically using the PS in all nine CHD neonates (Figure 4). The *SNR_sys_* of a systolic murmur in the CHD group was significantly higher than that in the control group (*p* < 0.01; Figure 5). Indeed, the *SNR_sys_* does not necessarily represent systolic murmurs directly, but merely indicates the relative amplitude of the PS outputs during systole in reference to diastole. In the present study, we enrolled patients with CHD whose diastolic murmurs were not auscultated. Moreover, S1 and S2 were identified with the use of simultaneously recorded ECG, and the systole was identified as the period between the preceding S1 and the following S2. Thus, elevated *SNR_sys_* implied vibration from the chest wall of the patients synchronized with the systolic period, most likely due to a systolic murmur.

**Figure 6:**
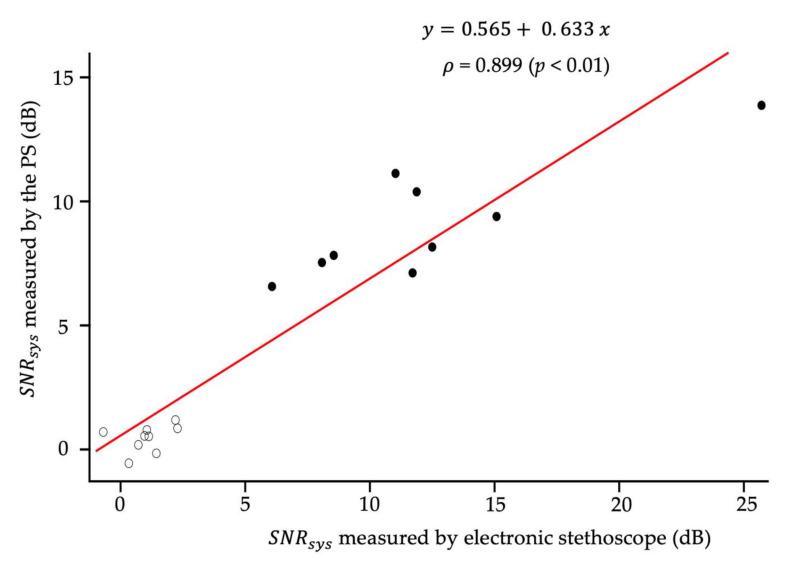
Scatter plot, linear regression line, and Spearman’s correlation coefficient between the signal-to-noise ratio (*SNR_sys_*) of a systolic murmur recorded by the piezoelectric sensor and that recorded by an electronic stethoscope (n = 18). Black circle indicates the *SNR_sys_* of the congenital heart defects (CHDs) group, while the white circle shows the *SNR_sys_* of the control group. The red line indicates the regression line.

The *SNR_sys_* was compared after using the PS and the electronic stethoscope. Figure 6 shows the relationship between *SNR_sys_* recorded by electronic stethoscope and the PS. In the control group (open symbols), the values of SNR were near 0 using the PS and stethoscope, indicating that the systolic murmur was hardly present. In contrast, the SNR of the CHD group ranged from 5 to 25. Therefore, the *SNR_sys_* of PS had a strong correlation with that recorded by an electronic stethoscope (ρ = 0.899 and *p* < 0.01, respectively; Figure 6). Considering that the slope was less than 1, these results suggested that the PS successfully recorded the systolic heart murmur with a relatively lower sensitivity compared with the electronic stethoscope. There was no significant change between the supine and prone positions in heart rate (138.7 ± 11.4 bpm vs. 137.9 ± 12.6 bpm, *p* = 0.751) and oxygen saturation (96.7 ± 3.1% vs. 96.9 ± 3.1%, *p* = 0.381).

## 4. Discussion

In this study, we successfully detected pathologic heart murmurs in neonates with CHDs using a PS. PS have some advantages over manual cardiac auscultation. PS can detect heart murmurs objectively. In contrast, heart murmurs detected by manual auscultation are subjective as they can differ depending on the listening ability of the physicians. Because the PS can display heartbeats and heart murmurs graphically with signal processing (Figure 5), individual errors can be smaller than those that occur during cardiac auscultation. Another advantage of the PS is that it can be easily used in neonates, which can reduce individual errors. When a PS is used along with routine medical examination, missed diagnoses of CHDs can be prevented. These advantages may be common to both PS and electronic stethoscopes. However, while the PS shown in this study is simply placed on the bed and detects vibrations from the entire chest wall, the electronic stethoscope is different, in that it can record heart sounds from various parts of the chest wall. Thus, electronic stethoscopes would be suitable for specialists to record their auscultation content, while the PS would be limited to screening purposes.

Another advantage of PS over manual cardiac auscultation or the use of electronic stethoscopes is that these sensors can detect vibrations without contact with the skin of neonates, which helps maintain comfort during recording and eliminates the risk of skin damage [9,10,11,12]. This is beneficial for premature infants who often have skin damage due to the fixation of ECG electrodes [22]. Furthermore, as the PS has been developed using only piezoceramic elements and brass and plastic plates, it is safe, durable, easy to sterilize [11], and manufactured at a low cost. Thus, it is easy to use this device in small obstetric facilities where there is a lack of physicians with skills in pediatric cardiac examinations. 

It was shown that the sensitivity of PS sensor for detecting the systolic murmur was slightly lower than that of the stethoscope at the cut-off frequency of 70 Hz. The lower sensitivity seems more marked when the cut-off frequency further increased (Figure 3). This is probably because the sensor was placed under a face towel to avoid direct contact of the PS with the skin of the neonate. Further, the position of the sensor was not necessarily most favorable in every experiment, and a slight displacement from the best monitoring position may have attenuated the sensitivity. Nevertheless, the present results indicated that the PS can successfully detect the systolic murmur with the use of the optimum cut-off frequency.

However, our study had some limitations. First, the number of subjects enrolled in this study was very low; indeed, only nine neonates who had CHDs with systolic murmurs and nine healthy neonates without any heart murmurs were included. We did not include patients with CHDs whose heart murmurs were faint (< Levine II/VI systolic murmur) and, thus, it remains unclear how to detect the minimum threshold of vibration that the PS can detect as heart murmurs. Moreover, we did not assess healthy neonates with physiological or innocent heart murmurs. Heart murmurs are not rare in healthy neonates without CHDs [23,24]. To improve the accuracy of the PS while screening CHDs, it is necessary to differentiate the pathologic heart murmurs from the physiological ones. Thus, the present study is considered a preliminary study. Further studies with larger sample sizes including patients with CHD and healthy neonates with faint heart murmurs, respectively, should be conducted to assess the effectiveness and practicality of the PS.

Another limitation was the time and effort required for the procedure. In this study, the PS data were stored in computers, filtered automatically, and analyzed manually. The whole procedure took 10–20 min. To overcome this limitation, an automatic analyzing system needs to be developed to effortlessly detect heart murmurs within minutes. Finally, the PS used in this study could not detect heart murmurs in the supine position. The PS needs to face the anterior chest wall to detect vibrations caused by abnormal blood flow. Hence, further improvement of the PS is needed to detect heart murmurs in various positions.

## 5. Conclusions

In conclusion, the PS can noninvasively detect pathologic heart murmurs in newborns. To improve the accuracy of PS screening, further experiments for the detection of faint heart murmurs and mechanical improvement of the PS device are needed to shorten the time of measurement and minimize artifacts. By applying a new approach, such as an artificial neural network, to discriminate between physiologic and pathologic heart murmurs automatically [13,25,26,27], the PS can become a useful automatic screening device to detect pathologic heart murmurs.

## Figures and Tables

**Table 1:** Clinical characteristics of patients.

	CHD Group (n = 9)	Control Group (n = 9)
Male sex	0	5
Gestational age at birth (wks)	38.4 × (37.0–39.4)	36.6 × (32.1–40.3)
Birth weight (g)	2553 × (1997–3148)	2278 × (1889–3481)
Degree of murmur		
2	5	
3	4	
Echo diagnosis		
VSD	6	
TOF	1	
PA-VSD	1	
Single ventricle	1	
Prenatal diagnosis	2	
Age at measurement (days)	8 × (1–26)	12 × (3–26)

Data are presented as medians (ranges) or numbers. The degree of heart murmur was graded according to the Levine grading scale. CHD: congenital heart defect; VSD: ventricular septal defect; TOF: tetralogy of Fallot. PA-VSD: pulmonary atresia with ventricular septal defect.

## Data Availability

Not applicable.
